# Parallel Reaction Monitoring: A Targeted Experiment Performed Using High Resolution and High Mass Accuracy Mass Spectrometry

**DOI:** 10.3390/ijms161226120

**Published:** 2015-12-02

**Authors:** Navin Rauniyar

**Affiliations:** 1 W.M. Keck Foundation Biotechnology Resource Laboratory, School of Medicine, Yale University, 300 George Street, New Haven, CT 06511, USA; navin.rauniyar@yale.edu; Tel.: +1-203-785-6171; Fax: +1-203-737-2638; 2 Department of Molecular Biophysics & Biochemistry, Yale University, New Haven, CT 06520, USA

**Keywords:** parallel reaction monitoring, PRM, selected reaction monitoring, SRM, proteomics, mass spectrometry, targeted, quantitative proteomics, Q-Exactive, high resolution, accurate mass

## Abstract

The parallel reaction monitoring (PRM) assay has emerged as an alternative method of targeted quantification. The PRM assay is performed in a high resolution and high mass accuracy mode on a mass spectrometer. This review presents the features that make PRM a highly specific and selective method for targeted quantification using quadrupole-Orbitrap hybrid instruments. In addition, this review discusses the label-based and label-free methods of quantification that can be performed with the targeted approach.

## 1. Introduction

In discovery-based proteomics experiments, proteins that are specific to disease and treatment conditions, or are potential biomarker candidates, are identified in an unbiased manner. Even though discovery-based proteomics by data-dependent acquisition is a powerful technique for unbiased identification of proteins in a sample, it is less effective for the consistent detection of protein of interest due to the stochastic nature of data acquisition. However, for quantification purposes, it is imperative to consistently detect proteins of interest across samples in order to obtain sufficient statistical power to establish the association of proteins or biomarkers with the disease in question. Nevertheless, data-dependent acquisition provides the key information required to build subsequent targeted experiments. This information includes identity of protein, amino acid sequence, retention time and charge state of peptides, and the distribution and intensity of the fragment ions in the MS/MS spectrum.

In a targeted experimental workflow, selected peptides that are surrogates exclusively of proteins of interest are measured in a predefined *m*/*z* ranges and retention time window. Targeted experiments are primarily performed on a triple-quadrupole (QQQ) and hybrid quadrupole-linear ion trap (QTrap) mass spectrometers using a data acquisition method known as Selected Reaction Monitoring (SRM) [1,2]. Recent studies have shown that targeted experiments known as Parallel Reaction Monitoring (PRM) can also be performed on hybrid quadrupole-Orbitrap (q-OT) and quadrupole time-of-flight (q-TOF) mass spectrometers [3,4,5,6,7,8]. The Orbitrap in a q-OT replaces the third quadrupole (Q3) mass analyzer of a QQQ [9]. Studies using SRM and PRM have shown that both these targeted methods have comparable sensitivity with similar linearity, dynamic range, precision, and repeatability for quantification of proteins [4,10,11,12]. However, PRM has certain advantages over SRM, such as it is relatively easier to build the data acquisition method because a priori selection of target transitions is not required. Furthermore, PRM provides high specificity because the MS/MS data is acquired in high resolution mode that can separate co-isolated background ions from the target peptide ions. In SRM, only three to five transitions are monitored, whereas in PRM a full MS/MS spectra is acquired that contains all the potential product ions and confirms identity of the target peptide.

## 2. Selected Reaction Monitoring (SRM)

When SRM-based targeted quantitative analysis is performed on a triple-quadrupole (QQQ) mass spectrometer, a predefined precursor ion is selected in the first quadrupole (Q1), then fragmented in the second quadrupole (Q2) that serves as a collision cell. After fragmentation, a predefined set of fragment ions (also known as transitions) are filtered in the third quadrupole (Q3) and these are then transmitted to the detector. The peak area of each of the transitions of a precursor ion is integrated and used for quantification. The integrated peak areas support relative or, if known concentration of heavy isotope-labeled internal standards are spiked in the sample, absolute quantification of the targeted peptides.

The first and third quadrupoles (Q1 and Q3) in a triple-quadrupole mass spectrometer are mass analyzers that act as two mass filters, one for the precursor ion and the other for the fragment ions. Due to the two levels of mass filtering, most of the co-eluting interferences are effectively excluded, making SRM a highly sensitive technique. However, the Q1 and Q3 mass analyzers have low resolving power, so they cannot separate interfering near-isobaric ions that co-elute with the target peptides [13]. In complex matrix, when these co-eluting peptides are present in high abundance and have similar MS/MS fragmentation patterns, they interfere with the signal from the target peptide. Since in SRM only a limited number of transitions are monitored for each peptide, such interferences may confound the data analysis.

Furthermore, SRM requires significant effort in building a data acquisition method for a newer set of candidate proteins [14]. In addition to the *m*/*z* of the peptides, it is necessary to know beforehand the fragment ions to target [15]. Also, in order to select the best set of peptides and transitions for the candidate proteins, multiple iterations and optimizations may be required [14]. Thus, the success of SRM is dependent on the transitions of the target peptides that are pre-selected and used for monitoring during data acquisition. Selecting the best possible transitions for the target proteins results in reliable quantification.

## 3. Parallel Reaction Monitoring (PRM)

PRM is a targeted method of quantification performed using high-resolution hybrid mass spectrometers such as quadrupole-Orbitrap (q-OT) [4,6,16]. The development and implementation of higher energy collision-induced dissociation (HCD) fragmentation enabled MS/MS spectra to be acquired in the Orbitrap analyzer with high resolution and high mass accuracy. HCD is a beam-type collisional dissociation similar to the dissociation achieved in triple quadrupole mass spectrometers, as well as in quadrupole time-of-flight (QTOF) mass spectrometers [17]. An advantage of the q-OT mass spectrometer is that both discovery and targeted experiments can be performed on the same instrument. This makes it easier to transfer instrumental parameters (e.g., collision energy, quadrupole isolation window, automatic gain control, retention time *etc.*) between the two data acquisition methods.

**Figure 1:**
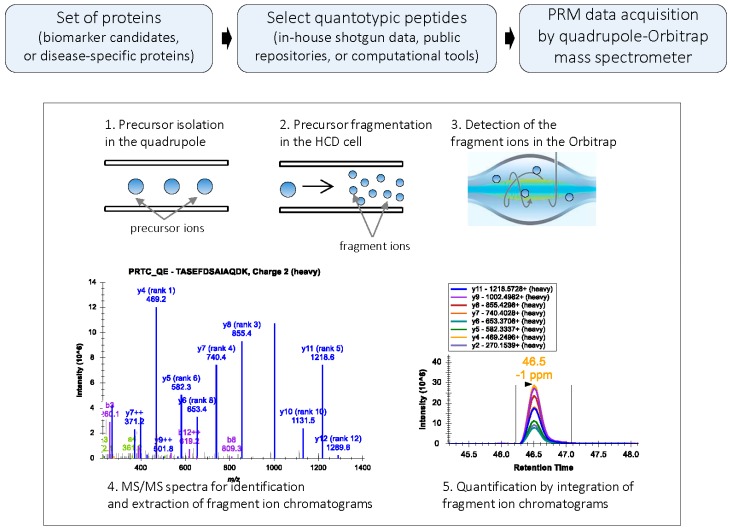
PRM workflow: In PRM, a target precursor ion is isolated in the quadrupole analyzer and fragmented in the HCD cell; the fragment ions are then detected in the Orbitrap mass analyzer. PRM generates high resolution, full MS/MS data. During data processing, the MS/MS spectrum is used for the confident identification of the peptide; subsets of fragment ions with highest intensities in the MS/MS spectrum are used for quantification. Peak areas of fragment ions are extracted using <10 ppm mass window and integrated across the elution profile. The Skyline platform supports PRM-based targeted MS quantification [20].

In PRM, when performed in q-OT, a predefined precursor ion is selected in the quadrupole and transferred via the C-trap to the HCD cell for fragmentation. The C-trap can fill with ions for longer times, increasing signal-to-noise ratio of the ions measured in the Orbitrap [4,18]. From the HCD cell, fragment ions are transferred back to the C-trap and eventually injected and analyzed in the Orbitrap mass analyzer (Figure 1). Since full MS/MS spectra of the targeted peptides are acquired with high resolution and high mass accuracy, a PRM-based targeted method of protein quantification is highly selective and specific [3,4]. To further improve data acquisition efficiency, isotopically-labeled internal standards can be used to drive PRM acquisitions of the endogenous peptides (IS-PRM) [19].

### 3.1. Applications of PRM

PRM-based targeted method has been successfully applied in the validation of relative abundance of proteins and their posttranslational modifications (PTMs).

#### 3.1.1. PRM in Validation of Protein Relative Abundance

PRM has been used to quantify proteins in biological fluids. In a study by Kim *et al*. [21], the PRM assay was developed to selectively measure isotypes of serum amyloid A (SAA), SAA1 and SAA2, protein. The isotype-specific PRM assays successfully measured five allelic variants (1α, 1β, 1γ, 2α, 2β) of SAA1 and SAA2 in plasma samples from lung cancer patients. Ronsein *et al*. [12] have developed a PRM method for quantification of the high-density lipoprotein (HDL) proteome in plasma samples using ^15^N-labeled apolipoprotein A-1 as an internal standard. PRM, thus, provided a new strategy for an accurate and sensitive quantification of HDL proteins in translational studies. In a study by Khristenko *et al*. [22], PRM method was used to investigate the variability of protein abundances in 56 urine samples, collected from six volunteers participating in the spaceflight simulation program. Out of 1586 target peptides, 82% were systematically identified across four replicates and reproducibly quantified.

The PRM-based targeted method is an efficient approach for samples that are available in limited amounts due to various reasons, such as low volumes available (e.g., mouse serum) or less blood is withdrawn to reduce the stress level in the experimental animals [23]. PRM was used to quantify hepcidin peptide in monkey and mouse sera treated with small interfering RNA (siRNA) that targets hepcidin [23]. Hepcidin levels are elevated in anemia of chronic disease (ACD), and to reduce the serum hepcidin levels, a promising therapeutic approach includes the use of siRNA. This study showed that siRNA effectively reduced the serum level of hepcidin. Interestingly, compared to SRM, the results from PRM analysis showed at least 10-fold improvements in specificity and sensitivity (significant reduction in background noise) and consumed less volume of serum samples.

PRM has been used to validate quantification results from discovery-based proteomics studies. The relative expression of desmin and filamin C (FLNC) peptides, obtained from skeletal muscle samples from desminopathy patients, was validated using PRM [24]. The PRM results were consistent with the relative quantification results obtained by label-free analysis, and validated the finding that desmin and FLNC are over-represented in desminopathy aggregates. In a study by Du *et al*. [25], PRM was applied to verify a subset of proteins that are differentially expressed when the proteome of equine monocyte-derived macrophages (eMDMs), uninfected and infected with pathogenic strain of equine infectious anemia virus (EIAV), were examined using the iTRAQ method. In this study, out of 210 differentially expressed proteins, expression levels of 10 proteins were selected for validation by PRM and successfully confirmed.

#### 3.1.2. PRM in Validation of Proteins Posttranslational Modifications (PTMs)

PRM can differentiate and quantify peptides with isobaric PTMs. In mouse embryonic fibroblasts (MEF) cells, in which histone H3 K36 trimethyltransferase SETD2 was knocked out, PRM was used to quantify acetyl and methyl modifications [26]. In the same study, PRM was also used to quantify histone modifications in human neural stem cells (hNSC) treated with vitamin C [26].

Changes in the abundance or the glycosylation of serum glycoproteins are associated with the severity of cancer and other diseases. PRM can be used for high throughput and reproducible quantification of *N*-linked glycosite-containing peptides from serum glycoproteins. In a study by Thomas *et al*. [7], PRM was used to measure the relative abundance of *N*-linked glycosite-containing peptides in serum from patients with negative, nonaggressive, and aggressive prostate cancer biopsies. Out of 41 *N*-linked glycosite-containing target peptides (corresponding to 37 proteins) selected for PRM, the relative levels of four peptides were significantly different between the nonaggressive and aggressive prostate cancer groups.

PRM is also useful to study PTMs that are low abundant and are challenging to identify and quantify by shotgun proteomics methods. For example, to examine the acute inflammation damage of diabetes, Yu *et al*. [27] used PRM to identify and quantify nitration level in α-oxoglutarate dehydrogenase (α-OGDH) isolated from myocardial tissue of healthy and diabetic mouse. Tsuchiya *et al*. [8] used PRM to quantify ubiquitylation levels of ubiquitin-proline-β-galactose (Ub-P-βgal), a substrate of the ubiquitin fusion degradation pathway. In this study, they showed that K29-linked ubiquitin chains are attached to Ub-P-βgal, thus highlighting the sensitivity of PRM in studying complicated ubiquitin system in biological samples.

Sweredoski *et al*. [28] have used the PRM assay in combination with the middle-down electron transfer dissociation (ETD) approach to quantify multiply charged peptides that are larger than 5 kDa. When analyzing large multiply charged peptides by data-dependent acquisition method (middle-down proteomics), multiple charge states of the same peptide are resampled, precluding the selection and fragmentation of other proteoforms. In contrast, PRM allows an in-depth analysis of all potential proteoforms present in the sample because only one predetermined charge state of a given peptide will be selected for fragmentation. In their study, Sweredoski *et al*. [28] have used histone H3 fractions from untreated and DMSO-treated Murine ErythroLeukemia (MEL) cells and identified combinatorial PTMs on 254 histone H3 N-terminal fragments.

### 3.2. Steps of a PRM Experiment

The PRM method is designed to quantitatively monitor selective endogenous proteins and spiked internal standards across biological samples [8,21]. In PRM, as in SRM, endogenous peptides that are quantifiable surrogates of protein of interest are first selected. The selected peptides should be specific and stoichiometric to the protein of interest [29]. Multiple MS/MS data points of the peptides are then acquired across the elution profile. The data is then used to determine the abundance level of peptides, and subsequently their corresponding proteins, in the samples.

#### 3.2.1. Criteria for Selecting Target Peptides

Regardless whether SRM or PRM is used to acquire the data, selecting true surrogate peptides for proteins of interest is crucial in a targeted approach. Only the correct set of peptides can yield reliable quantification of the selected proteins. Peptides that are always observed for a specific protein, regardless of whether they are suitable for quantification, are known as proteotypic peptides [30]. Proteotypic peptides are unique to a given protein; these peptides fragment well in a mass spectrometer such that they can be confidently identified [30]. Only the proteotypic peptides that are suitable for quantification are known as quantotypic peptides [29]. Not all proteotypic peptides are quantotypic, but all quantotypic peptides are proteotypic [31]. The key feature of a quantotypic peptide is that its abundance must correlate with the abundance of the parent protein. Presence of a modified form of a peptide will decrease the level of its unmodified form. Hence, targeting only the unmodified peptides of a protein in such cases will give erroneous protein abundance results.

**Figure 2:**
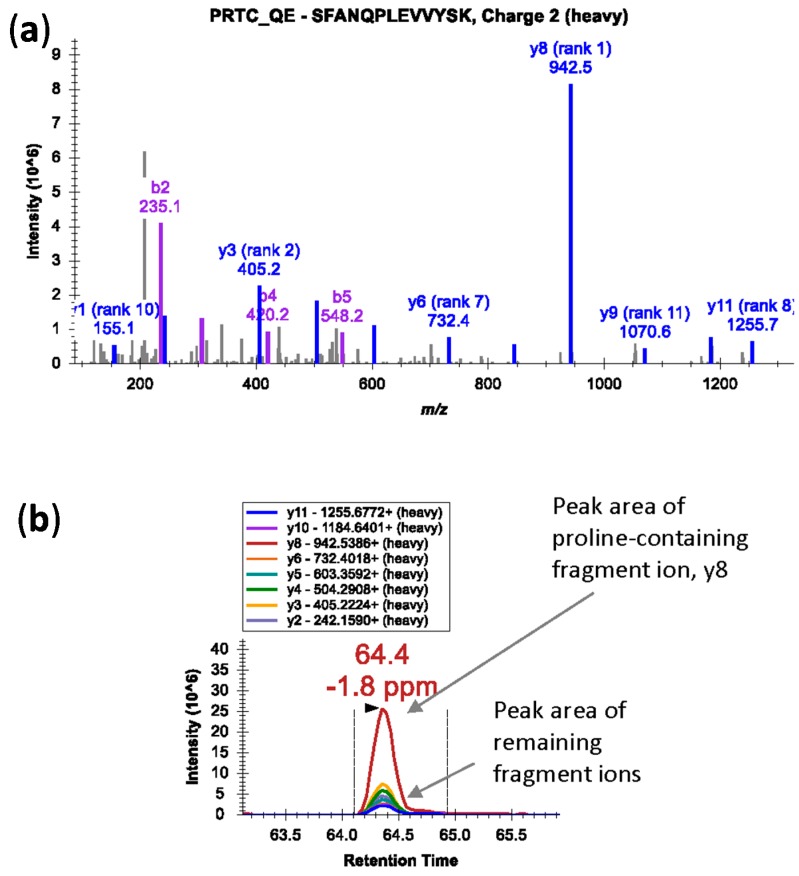
(**a**) An MS/MS spectrum of heavy isotope-labeled peptide SFANQPLEVVYSK* (*m*/*z* 745.3924, 2+) showing dominant y8 fragment ion by cleavage at N-terminal bond of proline residue; (**b**) Peak area contributions of the individual fragment ions of the peptide.

Criteria for selecting quantotypic target peptides are given below:

- Peptide length: *m*/*z* value of the peptides should be within the mass range of the instrument. Peptides of 8–25 amino acids are usually preferred.
- Uniqueness: Selected peptide should be unique to the proteins of interest. A search with the Basic Local Alignment Search Tool (BLAST) on the peptides can confirm if the peptide sequence is unique to the candidate protein.
- Miscleavage: When using trypsin, selected peptides should be fully tryptic and should not contain miscleavage sites. However, if proline is at the carboxylic side of arginine and lysine, the bond is resistant to trypsin cleavage [32]. Nevertheless, miscleavage at this site is considered reproducible and hence the peptides are suitable for targeted experiments [32]. Also, in case of proline-containing peptides, fragmentation at the N-terminal side of proline residue in a mass spectrometer generates an intense fragment ion; the fragment ion peak is dramatically over-represented in the MS/MS spectra (Figure 2a) [33]. In such cases, peak area contributions from other transitions can be significantly lower (Figure 2b). Avoid peptides with ragged ends—series of arginine and lysine amino acids, e.g., KK, KR, RK, or RR.
- Modification: Amino acid modifications can alter the cleavage pattern of peptides by the proteolytic enzyme. Therefore, avoid peptides that have amino acids susceptible to chemical modification during sample preparation. For example, methionine and tryptophan can undergo oxidation; glutamine and asparagine can undergo deamidation [34,35]. Both glutamate and glutamine at the N-termini can cyclize to pyroglutamate [36]. Also, avoid peptides with known posttranslational modification sites (e.g., phosphorylation (S/T), *N*-glycosylation (sequon N-*X*-S/T), or acetylation) unless the modified form is specifically targeted. In cases where the choice of peptides is limited, modified peptides may be considered if the rate of modification is reproducible and consistent across samples [35]. For example, cysteine-containing peptides are included as a target because reduction and alkylation of cysteine residues is usually a standard procedure in most proteomic workflow.
- Precursor charge: The peptide charge state that fragments better and generates the most sensitive measurements should be selected. Doubly or triply charged precursor ions are favorable due to their measureable *m*/*z* ranges. The observed charge state of a peptide may differ in a complex mixture compared with a purified sample. Histidine confers multiple charges to precursor and product ions, and hence should be avoided [32]. For example, under acidic conditions a tryptic peptide with a single histidine can be triply-charged instead of doubly-charged.
- Chromatographic peak: The shape of chromatographic peak of a peptide should be symmetrical with narrow width. If there is an option to select multiple peptides per protein, select peptides that have very different retention times.
- Signal intensity: The peptide should ionize efficiently and provide a stable and intense signal.

In the early stages of method development and optimization, multiple peptides per protein should be tested to find the correct set of surrogate peptides for proteins of interest. For the final quantitative analysis, a minimum of two peptides per protein should be selected; however, more peptides would provide robust quantification in the event of a discrepancy in quantitative results between two peptides [37].

The quantotypic peptides for targeted experiments can be selected from discovery-based experiments performed in the investigator’s own laboratory or searching publicly available databases, such as Global Proteome Machine (GPM) [38], PeptideAtlas [39], or Human Proteinpedia [40]. PeptideAtlas is a multi-species compendium of shotgun proteomic data provided by the scientific community [41]. It is a useful resource for planning targeted proteomics experiments. The data in PeptideAtlas contains information such as types of peptides and number of times they have been detected, predominant charge state, fragmentation pattern in MS/MS spectrum, and relative abundance of each fragment ions.

Computational algorithms can also be used for predicting quantotypic peptides. Some of the common computational algorithms are PeptideSieve [30], enhanced signature peptide (ESP) predictor [42], consensus predictor for Q-peptide sequence (CONSeQuence) [43], and peptide response predictor (PREGO) [44].

#### 3.2.2. Building a PRM Data Acquisition Method

A PRM method is relatively easy to set up because a full MS/MS spectrum is acquired for each target peptide [3].

(i) Existing discovery data from shotgun proteomic experiments speeds up the process of building targeted data acquisition methods [14]. Necessary information about the target peptides (e.g., *m*/*z* of the precursor ions, charge state, elution time) can be obtained from such data.
(ii) For reliable quantification, usually 8–10 scans across the chromatographic peak are recommended [45]. If an average width of a chromatogram is ~30 s, then cycle time must be adjusted to 3 s or less. Cycle time is the time it takes to cycle through the entire list of the target peptides. Cycle time is determined by the number of target peptides and their ion injection time. Ion injection time is synchronized with the Orbitrap resolution via the transient acquisition time [9]. Thus
Cycle time = Number of Peptides × Transient Length
(1)

**Figure 3:**
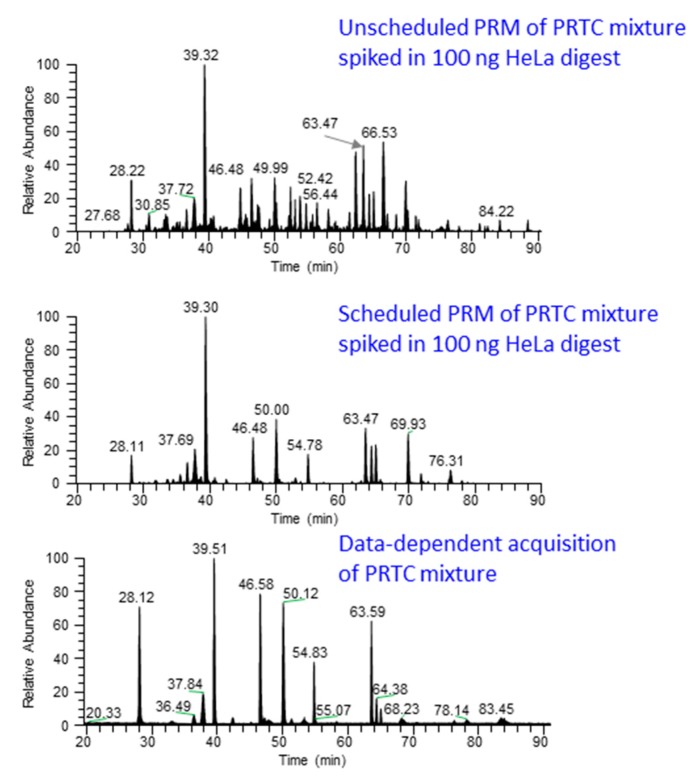
Comparison of scheduled and unscheduled PRM method. PRM analysis by scheduling the elution time of PRTC (Pierce Peptide Retention Time Calibration mixture, Catalog number: 88321) peptides spiked in 100 ng HeLa digest produces “cleaner” data (**middle** panel) compared to unscheduled PRM analysis (**top** panel). In the scheduled method, chromatographic peaks are only observed for the target peptides while unscheduled analysis contains noise peaks, possibly from the near-isobaric peptide ions. The **lower** panel shows the chromatographic profile from data-dependent acquisition of pure PRTC peptides.

Lower resolution is associated with shorter transient time, and is an effective way to analyze a larger number of peptides with enough data points [4]. However, lower resolution would reduce the selectivity, the main benefit of the PRM method [3].

(iii) The balance between cycle time for targeting a large number of peptides and high resolution can be achieved by scheduling the elution time window (start and end) of the peptides. Scheduling the elution time allows quantification of larger number of targets because the instrument acquires MS/MS of the peptide of interest only during an anticipated elution time interval [35,46]. Figure 3 shows the chromatograms from unscheduled and scheduled PRM methods.

For successful scheduling, reproducibility of peptide elution time is critical [11]. To account for drifts in peptide elution time due to various reasons such as fluctuation in column temperature, flow restriction through the column or electrospray ionization tip [46], relatively wider elution windows should be monitored; however, if windows are made too wide it will limit the total number of peptides that can be monitored per run [11,46]. To correct the drifts in the elution time, a set of “landmark” peptides that evenly distribute over the entire elution range can be added to the sample before acquiring the data [46].

(iv) Chromatographic gradients should be kept short as it results in sharper and higher peaks. The sharper and higher peak is, the higher the signal to noise and the better the quantification.

#### 3.2.3. Processing of PRM Data

(i) A spectral library with high quality reference MS/MS spectra for the target peptides is essential for the reliable results and success of PRM [4,47]. Since full MS/MS spectra are acquired in PRM, comparing the spectra with the annotated reference MS/MS spectra confirms the correct identity of the peptides [45]. Also, the MS/MS spectra from PRM can be searched directly with traditional database search engines.
(ii) In PRM, most fragment ions of a peptide can be used for quantification. However, for confident quantitative results, fragment ions with an *m*/*z* above the precursor *m*/*z* should be used because they are more selective [35]. These ions are less susceptible to interferences from co-eluting singly charged precursors because singly charged ions cannot result in fragments with a higher *m*/*z* than the precursor. In addition, the selected fragment ions should have high intensity [5]. Peptides are quantified by extracting peak areas of the qualified fragment ions using tight mass tolerances (typically 5–10 ppm). The ions that did not result from fragmentation of the peptide backbone (such as residual precursor ion, fragment ions with loss of water or ammonium groups), however, should not be used for extracting peak areas. The peak areas of the ions are then integrated across the elution profile and used for quantification of the peptides. Skyline is a freely-available tool for processing targeted data including PRM [20].
(iii) The automatically processed data should be manually verified and product ions with interferences should be removed. In cases where co-isolated background ions contaminate fragment ions selected for quantification, PRM provides the flexibility to select a different subset of fragment ions post data acquisition so that reliable quantification can be achieved [7]. Only the fragment ions showing symmetrical chromatographic shapes should be used for quantification. In addition, automatically integrated boundaries for the peaks should be adjusted if the software could not reliably determine the boundaries. The peak boundaries must be the same for the target peptide and the corresponding heavy-labeled peptide.

**Figure 4:**
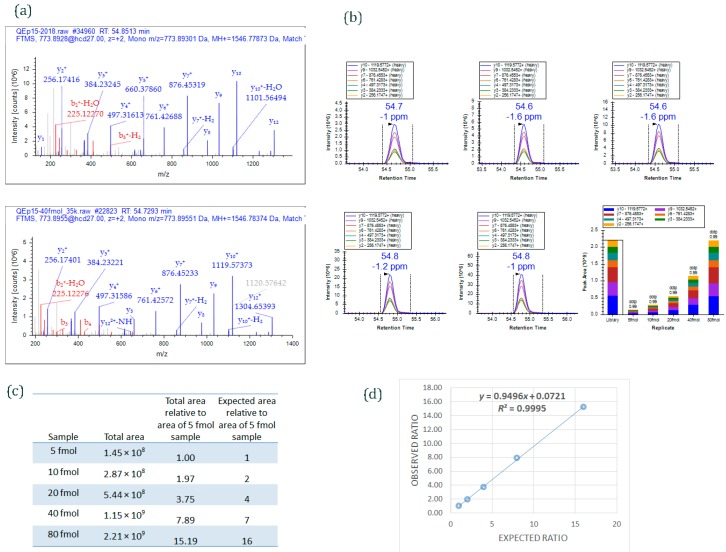
An example of data acquired by PRM method. Different amounts of a 15-peptide PRTC mixture (5, 10, 20, 40, and 80 fmol) was spiked into 100 ng HeLa digest and analyzed by PRM. (**a**) MS/MS spectrum of the heavy isotope labeled-peptide ELGQSGVDTYLQTK* (*m*/*z* 773.8955, 2+) acquired using data-dependent acquisition (**upper** panel) and used as an spectral library to match MS/MS spectrum of the peptide acquired by PRM method (**lower** panel); (**b**) Graphs displaying chromatograms of fragment ions extracted from the peptide ELGQSGVDTYLQTK (*m*/*z* 773.8955, 2+) at five different concentrations. Mass measurement error and retention time of the most intense transition is annotated above the peak. The vertical lines on either side of the peak indicate the integration boundaries for the peak. Bottom right plot: the total integrated fragment ion signal for the peptide at different concentrations is plotted as a bar graph; contribution from each individual fragment ion is displayed as a different color in the bars. Dot product (dotp) value indicates the degree of the match between spectral library MS/MS (Figure 4a) and the extracted ion chromatograms of the corresponding transitions [47]; high dotp indicates the absence of interfering signals [5]; (**c**) Total integrated area of the peaks of peptide ELGQSGVDTYLQTK at five different concentrations; (**d**) Scatter plot between expected ratios and observed ratios shows excellent correlation (*R*^2^ = 0.99). Skyline was used to analyze the PRM data.
(iv) As an example, Figure 4 shows data from a PRM experiment. The Thermo Scientific Pierce Peptide Retention Time Calibration (PRTC) mixture (Catalog #88321) contains 15 synthetic peptides that have ^15^N- and ^13^C-labeled arginine or lysine residues at C-terminal ends. Five different concentrations of PRTC (5, 10, 20, 40, and 80 fmol) were spiked into 100 ng HeLa digest (Pierce HeLa Protein Digest Standard, catalog #88329) and analyzed by PRM targeted method. Figure 4a shows MS/MS spectra of one of the heavy-labeled peptides from PRTC mixture, ELGQSGVDTYLQTK. The spectrum on the upper panel is from the peptide when PRTC (pure form) was run by data-dependent acquisition (DDA) mode; the spectrum on the lower panel is from the peptide when one of the concentrations of PRTC (40 fmol spiked into 100 ng HeLa digest) was run by PRM mode. The MS/MS spectrum from DDA analysis was used as a library spectrum. Figure 4b shows extracted ion chromatograms of fragment ions of ELGQSGVDTYLQTK peptide from 5, 10, 20, 40, and 80 fmol PRTC spiking experiments. The total peak area of the peptide observed at each spiking experiments is given in Figure 4c. An excellent correlation (*r* = 0.99) was obtained (Figure 4d) when observed ratios of the peptide was plotted against expected ratios. (The ratios were derived by comparing the total peak area of the peptide ELGQSGVDTYLQTK at different spiking level against the total area at 5 fmol.)

## 4. Quantification by Targeted Method

Targeted quantification methods can be label-based or label-free. In a targeted method (SRM or PRM), when heavy isotope-labeled peptides or full-length proteins are used for quantification of the endogenous proteins, the method is known as label-based. In label-based targeted quantification, endogenous peptides are quantified by comparing their signals with the internal standards that are spiked into the samples during processing. When heavy isotope-labeled peptides are not used as internal standards, the targeted method is known as label-free.

### 4.1. Internal Standards in Label-Based Targeted Quantification

Interferences from a complex matrix in the sample may suppress signals from target peptide ions or drift the sensitivity from run to run [48]. Therefore, targeted quantification requires internal standards to be spiked into the samples before acquiring the data. Internal standards reveal the technical variation of the experimental workflow and allow their correction during data analysis. Thus, internal standards enhance the accuracy and precision of the quantification. Internal standards also eliminate time-consuming *in vivo* or *in vitro* labeling of the samples and make it easier to transfer results between instruments and laboratories [49].

Most commonly used internal standards are heavy isotope-labeled synthetic peptides that are identical to the endogenous peptides. Heavy isotope-labeled full-length proteins can also be used as an internal standard. Modifications such as phosphorylation, methylation, and acetylation can be easily introduced into the heavy peptides; these modifications are, however, difficult to selectively introduce into heavy full-length proteins [50].

#### 4.1.1. Peptides as Internal Standards

Most commonly, heavy isotope-labeled synthetic peptides have arginine or lysine with ^13^C and ^15^N isotope at the C-terminal end. The heavy-labeled peptides have the same physicochemical properties as their endogenous counterparts; they have identical retention time, ionization efficiency, and MS/MS fragmentation pattern. However, they can be distinguished in a mass spectrometer based on the masses of the precursor and fragment ions.

During sample processing for targeted quantification using heavy peptides as internal standards, endogenous proteins are extracted from the samples and enzymatically digested. The resulting digests are spiked with heavy isotope-labeled peptides and the mixture is analyzed by the targeted liquid chromatography-mass spectrometry (LC-MS) method. However, the concentration of the endogenous target protein can vary from sample to sample. Therefore it is a challenge to determine the exact amount of the heavy-labeled peptides to spike. Excessive amounts of heavy peptides, compared to endogenous peptide, will saturate the detector [51,52]. In most cases, optimal amounts are determined experimentally by spiking different concentrations of the heavy-labeled peptides into the samples. The concentration of the heavy peptides selected for spiking is also based on the assumption that the efficiency of proteolytic digestion of the endogenous protein is 100% with complete recovery of the peptides throughout the sample processing steps. However, if the digestion is incomplete or peptide recovery is poor, then the level of the measured peptides may not be stoichiometric to the level of the protein. The use of Quantification concatamers (QconCAT) and heavy-labeled full-length proteins as internal standard addresses these issues to a certain extent [31,37,53].

#### 4.1.2. Proteins as Internal Standards

To normalize the variability in protein digestion, recovery, and mass spectrometry analysis, heavy-labeled full-length proteins such as QconCAT and protein standards for absolute quantification (PSAQ) are used as internal standards. QconCAT is an artificial protein designed as a linear concatenation of tryptic peptides [31]. It collectively provides target peptides for different proteins of interest [37]. PSAQ are heavy-labeled full-length proteins that are analogs of an endogenous target protein and are used as an internal standard [53,54,55]. Except for the mass, the full-length protein is identical to the endogenous protein. After enzymatic digestion, heavy-labeled peptides are released from QconCAT and PSAQ. The peptides are identical to the peptides released from the digestion of the corresponding endogenous protein. Thus, heavy-labeled full-length proteins in the form of QconCAT or PSAQ, when mixed with the sample in the beginning of the workflow, can reduce variability in measurements introduced during sample processing and data acquisition in a mass spectrometer.

### 4.2. Label-Based and Label-Free Targeted Quantification

As mentioned previously, targeted quantification method can be label-based or label-free.

#### 4.2.1. Label-Based Targeted Method

A label-based method can be used to determine the absolute and relative quantification of proteins in samples. Absolute quantification is essential for determining the stoichiometry of protein complexes, extent of posttranslational modification (e.g., phosphorylation), and concentration of protein biomarkers in body fluids. For absolute quantification, heavy isotope-labeled synthetic peptides, which are analogous to the targeted endogenous peptides, are spiked into the samples at known concentration [56]. The heavy and light peptides are both measured by the targeted approach. The absolute concentration of the endogenous peptides is then determined by comparing the sum of extracted peak area of transitions from the endogenous peptides with the sum of extracted peak area of transitions of the corresponding heavy-labeled peptides.

In label-based relative quantification, the precise concentration of heavy-labeled peptides is not known. Based on the purity level, the heavy-labeled peptides may allow an estimate of the amount of proteins present in the sample. When crude heavy-labeled peptides are spiked into the samples, ratios of peak areas of the light to heavy peptides across samples are used for relative quantification.

Ideally, for reliable quantification each endogenous peptide should have its own heavy isotope-labeled synthetic peptide. The co-eluting heavy peptides increase confidence in the identification and quantification of corresponding endogenous peptides. However, due to the high cost of the labeled peptides this may not be feasible in the initial method development phase, when large numbers of candidate peptides are screened. A cost-effective alternative strategy is to use a single heavy isotope-labeled peptide as a reference standard (labeled reference peptide, LRP) for quantification of all of the other endogenous target peptides [6,48]. Multiple heavy isotope-labeled reference peptides can also be used; these reference peptides should be chosen such that they are distributed across the entire chromatographic run, offering standards for early, medium and late-eluting peptides [6,48]. The multiple heavy isotope-labeled reference peptides can also be spiked at different concentrations in the samples, offering better quantification of high, medium, and low abundance peptides [48]. LRP-based quantification provides intermediate precision between using sequence-specific heavy isotope-labeled peptides and label-free analysis [48].

In addition to its use in quantification, labeled reference peptides can be used to evaluate the system performance and correct variation in chromatographic retention time shift between different LC runs [48].

#### 4.2.3. Label-Free Targeted Method

Label-free is a simple and straightforward method of targeted quantification. It is a cost-effective substitute for semi-quantitative measurements, and can be used when the difference between fold changes in proteins is greater than two fold [48,57]. In a label-free method, heavy isotope-labeled internal standards are not spiked, but an equal amount of samples are analyzed to estimate the relative abundance of proteins across samples [48]. Peak areas are extracted from the fragment ions, summed to generate peptide areas, and used for comparing it across the samples to determine the relative abundance of target proteins. The data obtained by a label-free targeted method can be normalized using peptides from housekeeping proteins whose abundance do not change from sample to sample [58].

For label-free analysis, the sample processing and LC-MS platform has to be highly reproducible, which is feasible by following standard operating and system maintenance procedures [58]. Quantification by a label-free targeted method yields confident results particularly in cases where substantially similar samples are analyzed [57,58]. Similar samples create similar ion suppression (if any) effects [58].

## 5. Sample Processing for Targeted Experiments

Even though targeted experiments are more sensitive than discovery-based experiments, high abundant proteins in the sample may interfere in the quantification of the low abundant target proteins. The interference is of serious concern particularly during quantification of potential biomarkers from serum and body fluids where the dynamic range of proteins can be very high [59]. For example, 85% of the total plasma protein consists of 5–10 very abundant proteins [60]. Hence, measures that reduce the sample complexity such as fractionation, enrichment [7], immunodepletion [60], or combinatorial peptide libraries [61] are applied to increase the limit of detection. Reducing the sample complexity overcomes ion suppression, limits interference of coeluting peptides, and maximizes the sensitivity of the targeted approach by eventually increasing the signal from the targets.

In addition to reducing sample complexity, for accurate and reliable quantification of proteins by targeted analysis, complete and consistent recovery of peptides from the sample is crucial [62,63]. Higher-order structure in native proteins hinders their complete digestion [64]. Incomplete digestion diminishes the ion current of peptides and presents an inaccurate level of endogenous protein in the sample [65]. Schmidt *et al*. [62] have shown that different denaturing conditions in the experimental workflow can produce different results on protein stoichiometry within protein complexes. Therefore, protein extraction buffer, protein precipitation methods, protein solubilization and digestion conditions should be evaluated before performing a targeted assay.

## 6. Conclusions

Targeted studies facilitate hypothesis-driven proteomic experiments by exclusively focusing on quantitative monitoring of predefined sets of proteins across multiple samples. The advent of quadrupole-Orbitrap mass spectrometers is progressively shifting the paradigm of targeted experiments from low resolution selected reaction monitoring (SRM) to high resolution parallel reaction monitoring (PRM). PRM is a novel, targeted quantification method performed in a high resolution and high mass accuracy mode on a quadrupole-Orbitrap mass spectrometer. Furthermore, continuous improvement in scan rate and resolving power in mass spectrometers is extending the capability of PRM to efficiently discriminate the targeted peptides from interfering background matrices and yield reliable quantification results. Increased scan rate increases the number of peptides analyzed in a PRM experiment while keeping the sensitivity and selectivity levels high. PRM has the potential to develop as an alternative to SRM for targeted quantification.

The application of PRM in various biological studies shows that it is a powerful targeted method for quantitative proteomics. It can be expected that its application will further expand and, in the future, may supplant the low-resolution targeted method. The popularity of PRM can be attributed to the simple and straightforward data acquisition method, and high selectivity and specificity because full MS/MS spectra of each target ions is acquired using high resolution and high mass accuracy.
